# Protective Effects of Rooibos (*Aspalathus linearis*) and/or Red Palm Oil (*Elaeis guineensis*) Supplementation on *tert*-Butyl Hydroperoxide-Induced Oxidative Hepatotoxicity in Wistar Rats

**DOI:** 10.1155/2013/984273

**Published:** 2013-04-18

**Authors:** Olawale R. Ajuwon, Emma Katengua-Thamahane, Jacques Van Rooyen, Oluwafemi O. Oguntibeju, Jeanine L. Marnewick

**Affiliations:** ^1^Oxidative Stress Research Centre, Department of Biomedical Sciences, Cape Peninsula University of Technology, P.O. Box 1906, Bellville 7535, South Africa; ^2^Experimental Antioxidant Research Laboratory, Department of Biomedical Sciences, Cape Peninsula University of Technology, P.O. Box 1906, Bellville 7535, South Africa

## Abstract

The possible protective effects of an aqueous rooibos extract (*Aspalathus linearis*), red palm oil (RPO) (*Elaeis guineensis*), or their combination on *tert*-butyl-hydroperoxide-(*t*-BHP-)induced oxidative hepatotoxicity in Wistar rats were investigated. *tert*-butyl hydroperoxide caused a significant (*P* < 0.05) elevation in conjugated dienes (CD) and malondialdehyde (MDA) levels, significantly (*P* < 0.05) decreased reduced glutathione (GSH) and GSH : GSSG ratio, and induced varying changes in activities of catalase, superoxide dismutase, glutathione peroxidase, and glutathione reductase in the blood and liver. This apparent oxidative injury was associated with histopathological changes in liver architecture and elevated levels of serum alanine aminotransferase (ALT), aspartate aminotransferase (AST), and lactate dehydrogenase (LDH). Supplementation with rooibos, RPO, or their combination significantly (*P* < 0.05) decreased CD and MDA levels in the liver and reduced serum level of ALT, AST, and LDH. Likewise, changes observed in the activities of antioxidant enzymes and impairment in redox status in the erythrocytes and liver were reversed. The observed protective effects when rooibos and RPO were supplemented concomitantly were neither additive nor synergistic. Our results suggested that rooibos and RPO, either supplemented alone or combined, are capable of alleviating *t*-BHP-induced oxidative hepatotoxicity, and the mechanism of this protection may involve inhibition of lipid peroxidation and modulation of antioxidants enzymes and glutathione status.

## 1. Introduction

The liver is a target organ for toxic substances because the hepatocytes that make up the majority of the liver structure are very active in the metabolism of xenobiotics. During detoxification of xenobiotics, reactive oxygen and nitrogen species (RONS) are generated which can result in oxidative or nitrosative stress. Both ROS and RNS are products of normal cellular metabolism and they may be deleterious or beneficial species. At low/moderate concentrations, ROS/RNS is involved in physiological roles including cell signalling, defence against infectious agents, and induction of mitogenic responses [1, 2]. However, overproduction of ROS arising from mitochondrial electron transport chain or excessive stimulation of NADPH results in oxidative stress, a deleterious process that can lead to damage to important cell structures, including lipids and membranes, proteins, and DNA [1, 3]. *tert*-butyl hydroperoxide (*t*-BHP) is a well-known oxidant that has been used as a model to investigate mechanisms of cellular damage caused by oxidative stress [4–7]. It can be metabolized to peroxyl and alkoxyl radicals by cytochrome P-450 in the hepatocytes, which in turn can initiate lipid peroxidation, producing loss of membrane fluidity, and mediating DNA damage [4, 8], which are known phenomena of oxidative stress in cells and/or tissues. Oxidative stress has been associated with cellular injury seen in many pathological conditions. In humans, oxidative stress is involved in many disease conditions, such as neurodegenerative disorders including Parkinson's and Alzheimer's disease, cardiovascular diseases, diabetes, and cancers [2, 3, 9]. Also evidence suggests that RONS are involved in the normal aging process as well as in age-related diseases [10].

Under normal circumstances several endogenous protective mechanisms have evolved in mammalian cells to limit free radicals and the damage caused by them. There are several antioxidant defense systems, including the action of antioxidant enzymes such as superoxide dismutase (SOD), catalase (CAT), and glutathione peroxidase (GPx) as well as nonenzymatic molecules, including reduced glutathione (GSH), ceruloplasmin, and transferrin [11, 12]. However, since this endogenous protection may not be sufficient when the formation of free radicals is excessive, especially during chronic disease conditions, additional protective mechanisms via dietary antioxidants are of great importance. The role of natural antioxidants, especially those derived from plants in modifying various health challenges is gaining a lot of attention with scientific evidence showing that vegetables, fruits, and teas have protective effects on and promote health [13–17]. The main factor that is probably responsible for these protective effects by fruits, vegetables, and teas is the high content of polyphenolic antioxidants which they contain [18, 19]. 

Rooibos (*Aspalathus linearis*) (Brum f) Dahlg. (Family Fabaceae; Tribe Crotalarieae) and red palm oil (RPO), from the fruit of the oil palm tree (*Elaeis guineensis*) Jacq. (Family Arecaceae), are two plant extracts exhibiting high antioxidant capacities. Rooibos herbal tea is made from the leaves and stems of the rooibos (*Aspalathus linearis*) plant, a shrubby legume that is indigenous to the Cederberg Mountains around Clamwilliam and its surrounding area, north of Cape Town in the Western Cape Province of South Africa. Its popularity as a health/functional beverage is increasing worldwide, partly because it is caffeine free [20], low in tannin content when compared to *Camellia sinensis* teas [21], and also because it is high in antioxidant and bioactive phytochemicals [22]. Polyphenolic constituents identified in rooibos include aspalathin (major polyphenol and unique only to rooibos), nothofagin, quercetin, rutin, isoquercitrin, orientin, luteolin, vitexin, and chrysoeriol [23, 24]. The antioxidant properties of rooibos have been confirmed both *in vitro* and *in vivo* [25–28]. Rooibos has been shown to be antimutagenic [29, 30], cancer modulating [31–33], anti-inflammatory [34], antidiabetic [35], cardioprotective [36], and modulating oxidative stress [27, 28, 37].

Red palm oil is a lipid extract from the fleshy orange-red mesocarp of the fruits of the oil palm tree. It is unique in that it contains an equal amount of saturated and unsaturated fatty acids, with about 44% palmitic acid, 5% stearic acid (both saturated), 40% oleic acid (monounsaturated), 10% linoleic acid, and 0.4%  *α*-linoleic acid (both polyunsaturated), with natural fat soluble tocopherol, tocotrienol, and carotenoids which may act as antioxidants [38, 39]. Apart from the fat soluble antioxidants found in palm oil, studies have shown that RPO also contains several phenolic compounds, including gallic, chlorogenic, gentisic, coumaric, and caffeic acids, as well as catechins, hesperidin, narirutin, and 4-hydroxyl benzoate, all of which have appreciable radical scavenging and antioxidant ability [40–42]. The health benefits of RPO have been highlighted in feeding experiments using different animal models. Red palm oil positively modulates the serum lipid profile when fed to experimental rats [43, 44]. Researchers have also reported on the protective effect of RPO in reducing oxidative stress [45] and being associated with better recovery and protection of hearts subjected to ischaemia/reperfusion injury [46–48].

Though several studies have investigated the health potential of rooibos and red palm oil individually, to the best of our knowledge, there has been no report of a comparative study on these two herbal extracts. It is against this background that we tested the hypothesis that rooibos in a commonly used concentration as consumed by humans and red palm oil would have a synergistically positive effect on biomarkers of oxidative stress and ameliorate hepatotoxicity induced by *t*-BHP in male Wistar rats.

## 2. Materials and Methods

### 2.1. Chemicals

The chemicals L-ascorbic acid, 2,2′-azobis (2-methylpropionamidine) dihydrochloride (AAPH), 2,2-azino-di-3-ethylbenzthiazoline sulfonate (ABTS), 5,5′-dithiobis-2-nitrobenzoic acid (DTNB), fluorescein sodium salt, formaldehyde, Folin Ciocalteu's phenol reagent, gallic acid, reduced glutathione (GSH), oxidized glutathione (GSSG), glutathione reductase (GR), hesperidin, histological grade formaldehyde, 6-hydroxydopamine, mangiferin, 1-methyl-2-vinylpyridinium trifluoromethanesulfonate (M2VP), *β*-nicotinamide adenine dinucleotide phosphate-reduced tetrasodium salt (NADPH), quercetin dihydrate, 6-hydroxy-2,5,7,8-tetramethylchroman-2-carboxylic acid (trolox), 2-thiobarbituric acid (TBA) and 2,4,6-tri[2-pyridyl]-s-triazine (TPTZ), iron chloride hexahydrate (FeCl_3_·6H_2_O), potassium persulfate, and *tert*-butyl hydroperoxide were obtained from Sigma-Aldrich (Johannesburg, South Africa). Diethylenetriaminepentaacetic acid (DETAPAC), 4-(dimethylamino)-cinnamaldehyde (DMACA) and malondialdehyde bis(diethyl acetal) (MDA), glacial acetic acid, trifluoroacetic acid, sulfuric acid (H_2_SO_4_), hexane, methanol (MeOH), ethanol (EtOH), dichloromethane, tetrahydrofuran, acetone, and hydrochloric acid (HCl) were purchased from Merck (Johannesburg, South Africa). All other reagents used were of analytical grade.

### 2.2. Plant Materials and Rooibos Herbal Tea Preparations

Fermented rooibos (superior grade) herbal tea was a generous gift from Rooibos Limited (Mr. Arend Redelinghuys, Clanwilliam, South Africa). Rooibos herbal tea was prepared at a concentration customarily used for tea making purposes [25]. An aqueous extract of rooibos (RTE) was prepared by the addition of freshly boiled tap water to tea leaves at a concentration of 2 g/100 mL. The mixture was allowed to stand at room temperature for 30 minutes with constant stirring, filtered, and dispensed into water bottles. The aqueous rooibos extract was fed to rats *ad libitum*, and fresh tea was prepared every second day. The RPO used in this study (Carotino baking fat) was supplied by Carotino SDN BHD (company number: 69046-T), Johar-Bahru, Malaysia. The rats were fed 200 *μ*L (equivalent to 7 g/kg diet) of the Carotino baking fat orally every day.

### 2.3. Animal Treatment and Experimental Design

Eighty, pathogen-free, male Wistar rats (240 ± 23 g) and standard rat pellets were obtained from the Primate Unit of Stellenbosch University (Tygerberg Campus, South Africa). The animals received humane care in accordance with the Principle of Laboratory Animal Care of the National Medical Research Council and the Guide for the Care and Use of Laboratory Animals of the National Academy of Sciences (National Institute of Health Publication no. 80-23, revised 1978). The protocol for the study was approved by CPUT's Faculty of Health and Wellness Sciences Research Ethics Committee (Ethics Certificate no.: CPUT/HAS-REC 2010/A003). The rats were housed individually in stainless steel wired-bottom cages fitted with polypropylene houses in an experimental animal holding facility kept at a temperature of between 22 and 24°C, with a 12 h light dark cycle and 50% humidity. The rats were fed standard rat pellets (SRP) *ad libitum* and had free access to tap water or the rooibos herbal tea extract. After acclimatization in the experimental animal holding facility for 1 week, the 80 animals were randomized into eight groups as shown in Table 1. Oxidative stress was induced by intraperitoneal (i.p.) injection of *t*-BHP (30 *μ*mol/100 g body weight) daily for the last two weeks of the 8-week study [49]. Fluid intake was monitored at an interval of 2 days for the duration of the study period. The general conditions of the rats were monitored daily throughout the study and body weights recorded weekly and at sacrifice. At the end of the experimental period, fasted (16 h) animals in all the groups were euthanized by i.p. injection of sodium pentobarbital (0.15 ml/100 g bw). About 8 ml of blood was collected via the abdominal aorta, and this was aliquoted into collection tubes with EDTA and without anticoagulant to obtain plasma and serum, respectively. Plasma/serum was separated immediately by centrifugation at 5 000 g for 5 min at 4°C. The liver was excised, washed twice with ice-cold PBS (10 mM phosphate buffered saline pH 7.2) to remove residual blood, blotted to dry, and weighed. A slice of the liver sample was taken and fixed in 10% buffered formaldehyde solution for histological examination. The remaining liver tissue was immediately frozen in liquid nitrogen and stored at −80°C for biochemical analyses.

### 2.4. Histopathological Examinations

Histopathological examinations were performed at the Department of Anatomy and Histology, Stellenbosch University (South Africa). Formalin-fixed liver tissues were embedded in paraffin and cut into sections (3–5 *μ*m thickness) and stained with haematoxylin and eosin (H&E). Examination of the stained tissue sections was done by a pathologist, who was blinded to the protocol of the study. 

### 2.5. Preparation of Soluble Liver Fraction

A 10% (w/v) homogenate of liver tissue was prepared in 50 mM NaH_2_PO_4_ containing 1 mM EDTA and 0.5% Triton-X (pH 7.5) and centrifuged at 10 000 g for 10 min at 4°C. The supernatant was collected and stored at −80°C until used for analyses of antioxidant capacities, lipid peroxidation, activity of antioxidant enzymes, and glutathione redox status. Protein content of samples (erythrocyte and liver homogenate) was determined using the BCA protein assay kit supplied by Pierce (IL, USA).

### 2.6. Soluble Solids, Total Polyphenols, Flavonol, and Flavanol Content Determination

The soluble solids content of the rooibos extract was determined gravimetrically (fifteen repetitions) after drying a 1 mL aliquot of the extract at 70°C for 24 hours. The total polyphenol content of the aqueous rooibos extracts was determined using the Folin Ciocalteu's phenol reagent according to the method described by Singleton et al. [50]. Briefly, 125 *μ*L of 0.2 N Folin reagent and 100 *μ*L of 7.5% Na_2_CO_3_ were added to 25 *μ*L of aqueous rooibos extract in a clear 96-well plate. The mixture was allowed to stand at room temperature for 2 hr and absorbance read at 765 nm in a Multiskan Spectrum plate reader (Thermo Fisher scientific, Waltham, MA, USA). Results were expressed as mg gallic acid equivalents/mg soluble solids. The flavanol content of the aqueous rooibos extract was determined colorimetrically at 640 nm using *p*-dimethylaminocinnamaldehyde (DMACA) according to the method of Treutter [51], and the results were expressed as mg catechin equivalents/mg soluble solids. The flavonol/flavones content was determined spectrophotometrically at 360 nm, and the results were expressed as mg quercetin equivalents/mg soluble solids [52].

### 2.7. Determination of Antioxidant Capacity

#### 2.7.1. Oxygen Radical Absorbance Capacity (ORAC) Assay

Subsamples of plasma and liver homogenates were first deproteinized using 0.5 M perchloric acid (1 : 1, v/v), and centrifuged at 10 000 g for 10 min and the resultant supernatant stored at −80°C prior to analysis [53]. The ORAC of the rooibos extract and protein-free samples of plasma and liver was determined according to a fluorometric method described by Ou et al. [54]. The reaction mixture consisted of 12 *μ*L of diluted protein-free sample (1 : 10 with 75 mM phosphate buffer, pH 7.4) and 138 *μ*L of fluorescein (14 *μ*M) which was used as a target for free radical attack. The reaction was initiated by the addition of 50 *μ*L AAPH (4.8 mM) and the fluorescence (excitation 485, emission 538) recorded every 5 min for 2 hr in a Fluoroskan Ascent plate reader (Thermo Fisher Scientific, Waltham, MA, USA). The ORAC values were calculated using regression equation *y* = *ax* ^2^ + *bx* + *c* between Trolox concentration (*μ*M) and the area under the curve. Results were expressed as *μ*M Trolox equivalents (TE)/L or *μ*M Trolox equivalents (TE)/g tissue.

#### 2.7.2. Trolox Equivalent Antioxidant Capacity (TEAC) Assay

The trolox equivalent antioxidant capacity of the aqueous rooibos extract was determined according to the method described by Re et al. [55]. The ABTS^•+^ solution was prepared 24 h before use by mixing ABTS salt (8 mM) with potassium peroxodisulfate (140 mM), and the solution stored in the dark until the assay could be performed. The ABTS^•+^ solution was diluted 1 : 20 with distilled water to give an absorbance of 1.50 at 734 nm. Each sample (25 *μ*L) was mixed with 275 *μ*L ABTS^+^ solution in a 96-well clear plate. The plate was read after 30 min incubation at room temperature in a Multiskan Spectrum plate reader (Thermo Fisher Scientific, Waltham, MA, USA). Trolox was used as the standard and results were expressed as *μ*M TE/L or *μ*M TE/g tissue.

#### 2.7.3. Ferric Reducing Ability of the Plasma (FRAP) Assay

The ferric reducing ability of the rooibos extract, plasma, and liver samples was determined using the method described by [56]. Briefly, 10 *μ*L of sample was mixed with 300 *μ*L FRAP reagent in a 96-well clear plate. The FRAP reagent was a mixture (10 : 1 : 1, v/v/v) of acetate buffer (300 mM, pH 3.6), TPTZ (10 mM in 100 mM HCl), and FeCl_3_·6H_2_O (20 mM). After incubation at room temperature for 30 min, the plate was read at a wavelength of 593 nm in a Multiskan Spectrum plate reader (Thermo Fisher Scientific, Waltham, MA, USA). Ascorbic acid (AA) was used as the standard and the results were expressed as *μ*mol AAE/L or *μ*mol AAE/g tissue.

### 2.8. High-Performance Liquid Chromatography Analysis of Aqueous Rooibos Extract

The rooibos tea extract was filtered (Whatman no 4) and chromatographically separated on an Agilent Technologies 1200 series HPLC system according to an adapted method described by Bramati et al. [24]. The HPLC system consisted of a G1315C diode array and multiple wavelengths detector, a G1311A quaternary pump, a G1329A autosampler, and a G1322A degasser. A 5 *μ*m YMC-Pack Pro C18 (150 mm × 4.6 mm i.d.) column was used for separation, and acquisition was set at 287 nm for aspalathin and 360 nm for other components. The mobile phases consisted of water (A) containing 300 *μ*L/L trifluoroacetic acid and methanol (B) containing 300 *μ*L/L trifluoroacetic acid. The gradient elution started at 95% (A) changing to 75% (A) after 5 min and to 20% (A) after 25 min and back to 95% (A) after 28 min. The flow rate was set at 0.8 mL/min, the injection volume was 20 *μ*L, and the column temperature was set at 23°C. Peaks were identified based on the retention time of the standards and confirmed by comparison of the wavelength scan spectra (set between 210 nm and 400 nm).

### 2.9. High-Performance Liquid Chromatography Analysis of RPO

#### 2.9.1. Vitamin E Content of RPO

Vitamin E in RPO was extracted by shaking 1 g of RPO in 5 mL of absolute ethanol for 30 min, followed by centrifugation at 3500 g for 10 min. The top vitamin E layer was analyzed on an Agilent Technology 1200 series HPLC system with the visible wavelength detector set at 296 nm. Twenty microlitre of sample was injected into the column (YMC-Pack Pro C18, 150 × 4.6 mm i.d., room temperature) and elution performed with a mobile phase consisting of (A) (acetonitrile : methanol : isopropanol : water;  45 : 45 : 5 : 5,  v/v) and (B) (acetonitrile : methanol : isopropanol; 50 : 45 : 5, v/v) at a flow rate of 1 mL/min. Mobile phase (A) was programmed to (B) within 10 min, and this condition was maintained for another 15 min before returning to the original conditions. The contents of tocopherols and tocotrienols were quantified by comparing the retention time and/or peak area with standards [57].

#### 2.9.2. Carotenoid Content of RPO

Carotenoids from RPO were extracted with tetrahydrofuran : dichloromethane (1 : 1, v/v) and analysed on an Agilent Technology 1200 series HPLC with the visible detector set at 450 nm according to a modified method of [58]. Twenty microlitre of extracted samples was injected automatically into the column (YMC-Pack Pro C30, 250 × 4.6 mm i.d., room temperature) and isocratic elution performed on a mobile phase consisting of methanol : acetone (9 : 1, v/v) with flow rate set at 1 mL/min. Peaks were identified based on the retention time of the *α*- and *β*-carotene standards.

### 2.10. Liver Function Tests

Serum alanine transaminase (ALT), aspartate transaminase (AST), and lactate dehydrogenase (LDH) were analysed using a Medica EasyRA automated clinical chemistry analyser (Medica Corporation Bedford, MA, USA) and standard diagnostic kits (Medica Corporation Bedford, MA, USA).

### 2.11. Oxidative Status Biomarkers

#### 2.11.1. Lipid Peroxidation

Lipid peroxidation was assessed by measurement of conjugated dienes (CDs) and malondialdehyde (MDA). Plasma and liver MDA were determined by HPLC using a method adapted from Khoschsorur et al. [59]. Briefly, plasma or liver homogenates (100 *μ*L) were mixed with orthophosphoric acid (0.44 M, 0.75 mL), aqueous TBA (42 mM, 0.25 mL), and water (twice distilled, 0.45 mL). The mixture was heated in a boiling water bath for 60 min. After cooling on ice, alkaline methanol (50 ml methanol + 4.5 ml 1 M NaOH) was added (1 : 1). The samples were centrifuged at 3 500 g for 3 min at 4°C. To 1 mL supernatant, 500 *μ*L of n-hexane was added and centrifuged at 15000 g for 40 sec and the supernatant collected. The neutralized reaction mixture (50 *μ*L) was then chromatographed on an Agilent 1200 series HPLC. A 5 *μ*m YMC-PackPro C18 (150 mm × 4.6 mm i.d.) column was used for separation with 60 : 40 (v/v) 50 mM phosphate buffer, pH 6.8-methanol as mobile phase. The flow rate was 1 mL min^−1^. Fluorometric detection was performed with excitation at 532 nm and emission at 552 nm. The peak of the MDA-TBA adduct was calibrated with an MDA standard processed in exactly the same way as the samples. Conjugated dienes were estimated according to the method of Recknagel and Glende [60]. Briefly, 405 *μ*L of chloroform-methanol mixture (2 : 1 v/v) was added to 100 *μ*L of plasma or liver homogenates. The mixture was vortexed for 60 s and centrifuged at 10 000 g for 10 min at 4°C. The top aqueous layer was discarded, and 200 *μ*L of the bottom chloroform layer was taken in a clean eppendorf tube and dried under nitrogen gas for 10 min. Cyclohexane (1 mL) was added to the tube and vortexed for 60 s. Two hundred microlitre of the mixture was taken into a clear 96-well plate, and the absorbance was read at 234 nm against a cyclohexane blank in a Multiskan Spectrum plate reader (Thermo Fisher scientific, USA). The concentration of CD was calculated according to the following equation:

(1) $$\begin{array}{c} \text{CD}\;=\frac{\left(A_{234s}-A_{234b}\right)}{\varepsilon }\times 10\;\left(\text{nmol}\;\text{CD}/\text{ml}\right), \end{array}$$

where *A*_234*s*_ is the absorbance of sample at 234 nm, *A*_234*b*_ is the absorbance of blank at 234 nm, and *ε* is the extinction coefficient = 2.95 × 10^4^.

#### 2.11.2. Antioxidant Enzyme Activity Assays

*Catalase. *Catalase (CAT) activity in the erythrocytes and liver homogenates were determined using the method described by [61]. In a clear 96-well plate, 5 *μ*L of sample and 170 *μ*L of 50 mM potassium phosphate, pH 7.0 was added followed by 50 *μ*L of 0.1% hydrogen peroxide in 50 mM potassium phosphate (pH 7.0) to initiate the reaction. The rate of decomposition of hydrogen peroxide was measured at 240 nm for 2 min in 15 s intervals in a Multiskan Spectrum plate reader (Thermo Fisher Scientific, USA). Catalase activity (*μ*mole H_2_O_2_ consumed/min/*μ*g protein) was determined using the molar extinction coefficient of 43.6 M^−1^ cm^−1^.

*Superoxide Dismutase*. The activity of superoxide dismutase (SOD) was determined according to the method of Crosti et al. [62]. The reaction mixture in a 96-well plate consisted of 15 *μ*L of sample, 170 *μ*L of 0.1 mM DETAPAC in 50 mM sodium phosphate buffer (pH 7.4), and 20 *μ*L of 1.6 mM 6- hydroxydopamine which initiated the reaction. The reaction was measured at 490 nm for 4 min at 30 s intervals and SOD activity expressed as U/mg of protein.

*Glutathione Peroxidase.* The activity of glutathione peroxidase (GPx) was determined according to the method of Ellerby and Bredesden [63] modified for a microplate reader. To the assay mixture containing 210 *μ*L of assay buffer (50 mM potassium phosphate, 1 mM EDTA pH 7.0), 2.5 *μ*L of GR (0.1 U/mL), 2.5 *μ*L of GSH (0.1 M), 5 *μ*L of NADPH (7.5 Mm), 2.5 *μ*L of sodium azide (100 mM), and 5 *μ*L of erythrocyte or liver homogenate, 25 *μ*L of H_2_O_2_ (15 mM) was added. The rate of H_2_O_2_-dependent oxidation of NADPH was immediately monitored at 340 nm for 2 min at 30 s intervals. The activity of GPx was calculated using the extinction coefficient of 0.00622 *μ*M^−1^ cm^−1^, and the results were expressed as nmol NADPH oxidized/min/*μ*g protein.

*Glutathione Reductase.* The activity of glutathione reductase (GR) was determined by a method of Staal et al. [64] modified for a microplate reader. Briefly, to the assay mixture containing 20 *μ*L of sample and 200 *μ*L of assay buffer (50 mM sodium phosphate and 25 mM of EDTA, pH 8.0), 20 *μ*L of 12.5 mM GSSG and 10 *μ*L of 3 mM NADPH were added. The rate of oxidation of NADPH was immediately monitored at 340 nm for 3 min at 30 s intervals. The activity of GR was calculated using the extinction coefficient of 0.00622 *μ*M^−1^ cm^−1^ and the results were expressed as *μ*mol NADPH oxidized/min/*μ*g protein.

#### 2.11.3. Glutathione Status Analysis

The total glutathione (GSH and GSSG) was measured according to the method described by Asensi et al. [65]. Aliquot of whole blood without (GSH) or with 3 mM freshly prepared M2VP (GSSG) were first deproteinized by 5% (w/v) metaphosphoric acid (MPA), while liver samples were homogenized (1 : 10) in 15% (w/v) TCA containing 1 mM EDTA for GSH determination and in 6% (v/v) PCA containing freshly prepared 3 mM M2VP and 1 mM EDTA for GSSG determination on ice. After centrifugation at 10 000 g for 10 min, 50 *μ*L of supernatant (from whole blood or liver homogenate) was added to 50 *μ*L of glutathione reductase (1 U) and 50 *μ*L of 0.3 mM DTNB. The reaction was initiated by addition of 1 mM NADPH to a final volume of 200 *μ*L. The change in absorbance was monitored at 410 nm for 5 min and levels calculated using pure GSH and GSSG as standards.

### 2.12. Statistical Analysis

Values were expressed as mean ± SEM. Data were tested for normality using the Kolmogorov-Smirnof Test and Levene's Test for Equality of variances. Differences between group means were estimated using one-way analysis of variance (ANOVA) followed by the Student-Newman-Keuls test for all pairwise comparisons. The Kruskal-Wallis Test, a nonparametric analogue to the one-way ANOVA, was used to test for group differences when data was not normally distributed. Results were considered statistically significant at *P* < 0.05 and marginally significant at *P* < 0.1. All the statistics were carried out using MedCalc v 12.2.1 software (MedCalc software bvba, Mariakerke, Belgium).

## 3. Results

### 3.1. Phenolic Content and Antioxidant Capacity of Aqueous Rooibos Tea Extract

**Figure 1:**
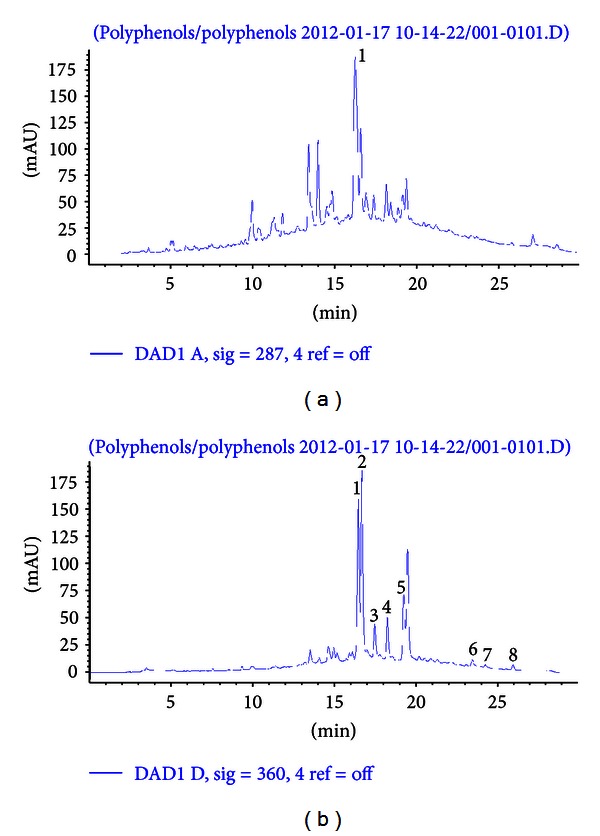
HPLC chromatogram of flavonoids in aqueous rooibos extract used in the study. (a) (287 nm), 1, aspalathin; (b) (360 nm), 1, orientin; 2, iso-orientin; 3, vitexin; 4, isovitexin; 5, hyperoside/rutin; 6, quercetin; 7, luteolin; 8, chrysoeriol.

Before the commencement of the study, the total phenolic content and *in vitro* antioxidant capacity of the rooibos extract were determined, and the results are shown in Table 2. The total phenolic content of the aqueous rooibos extract is 0.30 ± 0.01 mg GAE/mg soluble solid of which the flavonoids account for 68%.  Table 3 and Figure 1 show the HPLC quantification and daily intake of flavonoids in the rooibos extract. Aspalathin, isoorientin, and orientin were the major flavonoids consumed by the rats, with other notable flavonoids including vitexin, isovitexin, hyperoside/rutin, and trace amount of quercetin, luteolin, and chrysoeriol.

### 3.2. Fluid and Phenolic Intake of Rats Consuming the Aqueous Rooibos Extract

The fluid and phenolic intakes per day of the experimental rats consuming the rooibos extract are presented in Table 4. Water intake per day was similar between the negative and positive control rats. Rooibos intake across all groups consuming rooibos was also not different (*P* > 0.05), except for rats subjected to *t*-BHP injection and consuming a combination of rooibos and RPO that had a significantly lower (*P* < 0.05) daily fluid intake. As a result, the total phenolic and flavonoids intakes in this group of rats were also significantly lower (*P* < 0.05) when compared to the other groups consuming rooibos.

### 3.3. HPLC Quantification of Antioxidants in Red Palm Oil and Their Daily Intake

The different isomers of vitamin E and carotene quantified in the RPO used as well as their average daily intakes are shown in Table 5. Tocotrienols accounted for 80% of the vitamin E present in the RPO used in this study. *β*-Carotene accounted for 55% of the carotene, while *α*-carotene accounted for the remaining 45%.

### 3.4. Body and Liver Weight Changes

During the study, rats in all the experimental groups did not show any deleterious effects and no mortality was recorded. Estimated food intakes in all the treatment groups remained unchanged. The total body weight gain, absolute liver weight, and relative liver weight are shown in Table 6. The total body weight gain was lower in the positive control group compared to the negative control group, but the decrease was not significant (*P* > 0.05). Liver weights and relative liver weights were also similar in the positive and negative control groups. Rats consuming rooibos, RPO, or their combination without *t*-BHP injection maintained their body weights, liver weights and relative liver weights comparable (*P* > 0.05) to that of the negative control group, suggesting that rooibos and RPO had no adverse effects on the rats growth responses. The total body weight gain, liver weight, and relative liver weight of all *t*-BHP-treated rats consuming rooibos either alone or in combination with RPO were similar (*P* > 0.05) to those of the positive control. However, rats injected with *t*-BHP and consuming RPO alone had a significantly lower (*P* < 0.05) relative liver weight compared with the positive control rats.

### 3.5. Biochemical Markers of Liver Function

**Figure 2:**
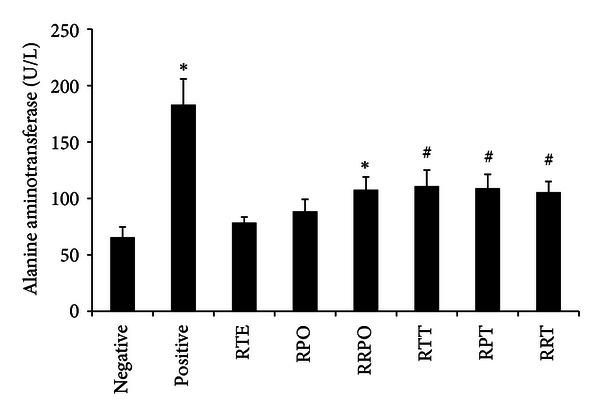
Effects of rooibos and RPO consumption on serum alanine aminotransferase (ALT) level in all experimental rats. Bars represent mean ± SEM of 7–10 rats. *Significantly different from negative control group (*P* < 0.05). ^#^Significantly different from positive control (*t*-BHP) group (*P* < 0.05). RTE: rooibos, RPO: red palm oil, RRPO: rooibos + RPO, RTT: rooibos + *t*-BHP, RPT: red palm oil + *t*-BHP, RRT: rooibos + red palm oil + *t*-BHP.

**Figure 3:**
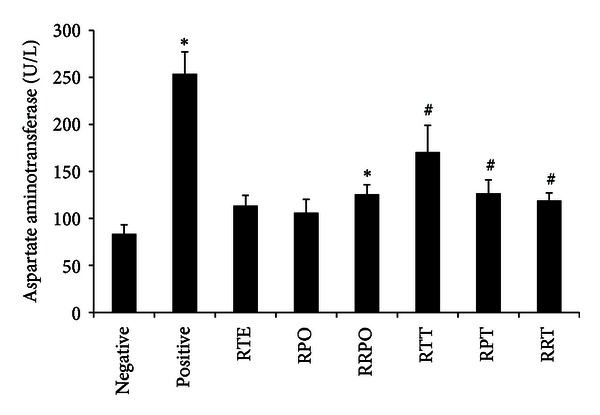
Effects of rooibos and RPO consumption on serum aspartate aminotransferase (AST) level in all experimental rats. Bars represent mean ± SEM of 7–10 rats. *Significantly different from negative control group (*P* < 0.05). ^#^Significantly different from positive control (*t*-BHP) group (*P* < 0.05). RTE: rooibos, RPO: red palm oil, RRPO: rooibos + RPO, RTT: rooibos + *t*-BHP, RPT: red palm oil + *t*-BHP, RRT: rooibos + red palm oil + *t*-BHP.

**Figure 4:**
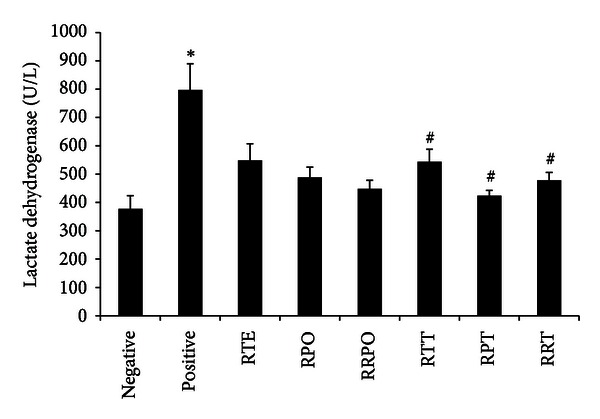
Effects of rooibos and RPO consumption on serum lactate dehydrogenase (LDH) level in all experimental rats. Bars represent mean ± SEM of 7–10 rats. *Significantly different from negative control group (*P* < 0.05). ^#^Significantly different from positive control (*t*-BHP) group (*P* < 0.05). RTE: rooibos, RPO: red palm oil, RRPO: rooibos + RPO, RTT: rooibos + *t*-BHP, RPT: red palm oil + *t*-BHP, RRT: rooibos + red palm oil + *t*-BHP.

The plasma hepatic marker enzyme levels of all treatment groups are presented in Figures 2, 3, and 4. Intraperitoneal injection of *t*-BHP for 2 weeks caused abnormal liver functions in treated rats. The level of plasma hepatospecific enzymes such as alanine amino transferase (ALT), aspartate transaminase (AST), and lactate dehydrogenase (LDH), was significantly increased (*P* < 0.05). *tert*-butyl hydroperoxide exposure brought about 2.79, 2.70, and 2.11-fold increase in the level of ALT, AST, and LDH, respectively, when compared to the negative control rats. Rooibos and RPO, when supplemented individually to rats without *t*-BHP treatment, did not have any significant effect (*P* > 0.05) on the level of ALT, AST, and LDH when compared with the negative control group. Upon oxidative stress induction with *t*-BHP, supplementation with rooibos extract significantly (*P* < 0.05) lowered the observed increases in ALT, AST and LDH by 39, 33, and 32%, respectively, while the reduction brought about by RPO constituted 40, 50, and 47%, respectively. Rats consuming a diet supplemented with RPO and rooibos as drinking fluid (RTE + RPO group), without *t*-BHP treatment showed a significant (*P* < 0.05) increase in ALT and AST levels when compared with negative control rats drinking water. However, when rooibos and RPO were supplemented simultaneously in *t*-BHP-exposed animals (RTE + RPO + *t*-BHP group), a significant decrease (*P* < 0.05) was observed for these liver marker enzymes (ALT, AST, and LDH) when compared to positive control rats.

### 3.6. Histopathological Observations

**Figure 5:**
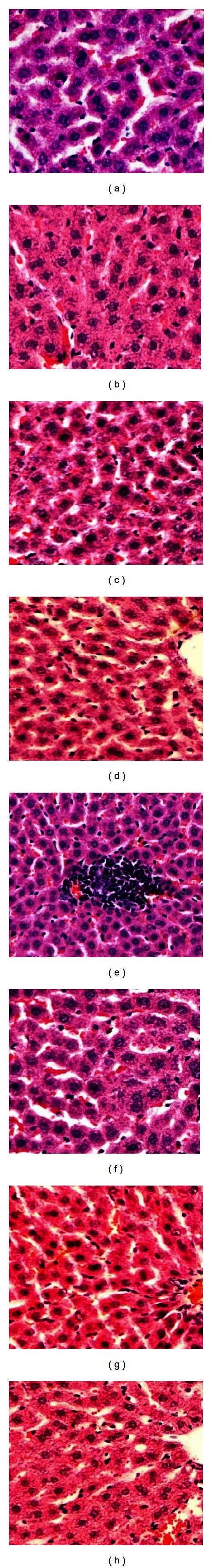
Histopathology of the liver showing (a)–(d) normal architecture with granulated cytoplasm and uniform nuclei of negative control, rooibos, RPO, or their combination, respectively, (H&E, ×20). (e) Positive control (*t*-BHP-treated rats) showing hepatocyte degeneration with massive lymphocyte and mononuclear cellular aggregation (H&E, ×20). (f)–(h) Rats pretreated with rooibos, RPO, or their combination before *t*-BHP treatment, showing almost normal hepatocellular architecture with slight lymphocyte infiltration (H&E, ×20).

Figure 5 shows the liver histoarchitecture of the different experimental groups, examined by conventional light microscopy in H&E stained sections.  Figure 5(a) shows the liver section of a negative control rat revealing normal architecture of hepatic cells with granulated cytoplasm and uniform nuclei. Rats consuming rooibos, RPO, or their combination without *t*-BHP treatment also exhibited normal histological architecture as shown in Figures 5(b)–5(d), respectively. Treatment with *t*-BHP resulted in alteration of liver histoarchitecture, evidenced by hepatocyte degeneration, with massive lymphocyte infiltration and mononuclear cell aggregation (Figure 5(e)). Rats consuming rooibos, RPO, or their combination with *t*-BHP treatment exhibited almost normal hepatocellular architecture, with slight lymphocyte infiltration (Figures 5(f)–5(h)).

### 3.7. Antioxidant Capacity of Plasma and Liver

The antioxidant capacity of plasma and liver samples was assessed as total polyphenol content, FRAP and ORAC activities (Table 7). Treatment with *t*-BHP resulted in a significant (*P* < 0.05) decrease in the level of plasma total polyphenols when compared to the rats consuming water (negative control). The consumption of the rooibos extract and RPO either alone or in combination did not restore these induced levels. Rats consuming the rooibos extract and RPO alone or in combination, without *t*-BHP treatment, also showed a significant (*P* < 0.05) decrease in their plasma levels of total polyphenols when compared to negative control rats. When considering the antioxidant capacity of the plasma, treatment with *t*-BHP caused a significant (*P* < 0.05) decrease in the ORAC values, but not in the FRAP values while cotreatment with rooibos alleviated this decrease and caused a significant (*P* < 0.05) enhancement of the plasma ORAC, with no such effect for RPO either alone or when combined with the rooibos extract. No significant differences were shown in the FRAP activity of plasma of rats consuming the rooibos, RPO, or combination (with/without *t*-BHP treatment). In the liver, *t*-BHP treatment resulted in a significant (*P* < 0.05) decrease in ORAC values but not in FRAP values. Co-treatment with rooibos, RPO, or their combination did not reverse the decrease. No significant differences were shown in the hepatic FRAP levels of rats consuming rooibos, RPO, or their combination, with or without *t*-BHP treatment. 

### 3.8. Antioxidant Enzyme Activity

The effects of aqueous rooibos extract, RPO, and/or their combination on antioxidant enzymes activities in erythrocytes and the liver of all experimental rats are presented in Table 8. In the erythrocyte, *t*-BHP treatment marginally (*P* < 0.1) increased the activity of CAT by about 36%, while the activities of GR, SOD, and GPx were significantly (*P* < 0.05) reduced by 63, 68, and 52%, respectively, when compared with the negative control rats. Rats consuming the rooibos extract, RPO, or their combination without *t*-BHP treatment had significantly (*P* < 0.05) decreased CAT activity, but showed no significant (*P* > 0.05) differences in the activities of GR, SOD, and GPx when compared with negative control rats. Consumption of rooibos, alone or in combination with RPO, reversed the changes induced by *t*-BHP by significantly (*P* < 0.05) lowering the CAT and increasing the GR, SOD and GPx activities compared with the positive control rats. Red palm oil consumption alone, by *t*-BHP-treated rats, significantly decreased CAT and increased GR and GPx activities, but showed no significant difference (*P* > 0.05) in the SOD activity when compared with the positive control rats.

Hepatic CAT and GPx were reduced marginally (*P* < 0.1) and significantly (*P* < 0.05), respectively, while GR activity was increased (*P* < 0.05) significantly, in the positive control group when compared with the negative control group. Rats consuming the rooibos extract or RPO without *t*-BHP treatment showed activities of hepatic CAT, GR, SOD and GPx comparable to those of the negative control rats, while the consumption of rooibos extract and RPO together in these rats significantly (*P* < 0.05) increased CAT and GPx activities when compared with the negative control rats. Consumption of the rooibos extract and RPO, either alone or in combination with *t*-BHP treatment, significantly (*P* < 0.05) increased GPx and decreased GR activity in the liver when compared with positive control rats. Only the combined supplementation of the rooibos extract and RPO was able to significantly (*P* < 0.05) increase CAT activity in these rats when compared with those of the positive control. The activity of SOD remained unchanged in the liver when *t*-BHP-treated rats (positive control) were compared with negative control rats, and also when *t*-BHP-treated rats consuming rooibos extract, RPO, or their combination were compared with the positive control rats. 

### 3.9. Lipid Peroxidation

The effects of the aqueous rooibos extract, RPO, or their combination on markers of lipid peroxidation in the plasma and liver of all experimental rats are presented in Table 9. In the plasma, the CD levels of *t*-BHP-treated rats (positive control) were significantly (*P* < 0.05) higher than those of the negative control rats; however, the MDA levels remained unchanged among these two groups. Rats consuming the rooibos extract without *t*-BHP treatment exhibited similar levels of CD and MDA when compared to rats consuming water (negative control). However, RPO alone or combined with rooibos without *t*-BHP treatment caused a significant (*P* < 0.05) increase in the level of conjugated dienes, but not MDA when compared to the negative control. Consuming the rooibos extract, RPO or their combination with *t*-BHP treatment significantly (*P* < 0.05) increased plasma CD levels, but MDA remain unchanged in these rats when compared with positive control rats.

In the liver, treatment with *t*-BHP resulted in a significant (*P* < 0.05) and marginal (*P* < 0.1) increase in CD and MDA, respectively, when compared to the rats consuming water (negative control). Rats consuming the rooibos extract, RPO, or their combination without *t*-BHP treatment exhibited similar levels of CD but a significantly lowered level of MDA when compared to the negative control rats. The increase in CD and MDA levels observed in the liver of *t*-BHP-treated rats was significantly (*P* < 0.05) reduced as a result of supplementation with rooibos extract, RPO, or their combination.

### 3.10. Glutathione Redox Status

The glutathione redox status of the different treatment groups is presented in Table 10. In the erythrocytes, the GSSG levels remained similar across all treatment groups. Treatment with *t*-BHP significantly (*P* < 0.05) depleted the GSH and resultant GSH/GSSG ratio by 70 and 75%, respectively, when compared to the negative control group. Rats consuming the rooibos extract or RPO alone without *t*-BHP treatment exhibited similar level of GSH and GSH/GSSG ratio when compared to rats consuming water (negative control). Cosupplementation of the rooibos extract and RPO in rats without *t*-BHP treatment significantly (*P* < 0.05) increased the GSH levels and GSH/GSSG ratio when compared to the negative control rats. Supplementation of rooibos extract, RPO, or their combination to *t*-BHP-treated rats was able to reverse the observed impairment in GSH redox status by significantly (*P* < 0.05) increasing the GSH levels and GSH/GSSG ratio to that comparable to levels found in rats drinking water (negative control).

Hepatic GSH level and GSH/GSSG ratio were significantly (*P* < 0.05) reduced, while GSSG remained unchanged in rats treated with *t*-BHP compared to negative control rats. Consumption of the rooibos extract alone, or combined with RPO, without *t*-BHP treatment significantly (*P* < 0.05) increased GSH level and GSH/GSSG ratio, but significantly (*P* < 0.05) decreased GSSG when compared to negative control rats. Rats consuming RPO alone, without *t*-BHP treatment exhibited similar GSH levels, but significantly (*P* < 0.05) decreased GSSG level and increased GSH/GSSG ratio when compared to the negative control animals consuming water. Supplementation of rooibos extract, RPO, or their combination to *t*-BHP-treated rats resulted in a significantly (*P* < 0.05) increased GSH level and GSH/GSSG ratio, parallel with a decreased GSSG level, when compared to positive control rats. The level of improvement observed in the redox status of this group of rats is comparable to what was obtained in negative control rats consuming water.

## 4. Discussion

Tert-butyl hydroperoxide is a membrane permanent prooxidant that has been extensively employed as a model for investigating the mechanism of cell injury initiated by oxidative stress in a variety of systems [4–6, 66]. Metabolism of *t*-BHP either by cytochrome P450 or haemoglobin triggers the generation of harmful free radicals such as alkoxyl and peroxyl radicals in the hepatocytes and erythrocytes. The free radicals readily cross cellular membranes and lead to formation of highly reactive hydroxyl radicals which can initiate lipid peroxidation, affect cell membrane integrity, damage protein, DNA, and result in cell injury in hepatocytes and rat liver [7, 67, 68]. An alternative metabolic pathway for *t*-BHP is its rapid conversion by GSH catalyzed by GPx to produce t-butanol and GSSG. The GSSG is then recycled back to GSH by the enzyme GR, resulting in NADPH oxidation. The depletion of GSH and the oxidation of NADPH are associated with Ca^2+^ homeostasis, a critical event in *t*-BHP-induced toxicity [6, 69].

A way of preventing free radical-mediated cellular injuries is to augment the oxidative defense capacity of the cell through intake of antioxidants. Recently, much attention has focused on the health beneficial role of naturally occurring antioxidants in biological systems. Phenolic phytochemicals derived from plants are being considered to play an important role as physiologically functional foods and are being utilized for treatment and prevention of clinical diseases related to oxidative stress, even though their modes of action may still not be fully understood [70]. The beneficial effects of these compounds are attributed to the antioxidant and free radical scavenging properties of their various components such as polyphenols and flavonoids [18, 19]. Rooibos (*Aspalathus linearis*) and red palm oil (RPO), from the fruit of the oil palm tree (*Elaeis guineensis*), are two such plant extracts exhibiting high antioxidant capacity. 

Rooibos is an important source of antioxidants due to its rich flavonoid content with numerous studies reporting on its health benefits. Its antioxidant [25, 71], anti-inflammatory [34], anti-diabetic [35], and abilities to modulate oxidative stress [27, 28, 37, 72] have been demonstrated in animal models and human studies. Red palm oil is rich in cocktail of lipid soluble antioxidants such as *α*- and *β*-carotene, lycopene, tocopherols (*α*, *β*, *γ*, and *δ* isoforms), tocotrienols (*α*, *β*, *γ*, and *δ* isoforms) and coenzyme Q_10_ [47, 73]. *In vivo* experiments using various animal models have revealed that RPO has many health benefits including protection against oxidative stress [44, 74], modulation of serum lipid profile [43, 44], and protection of the heart against ischaemia/reperfusion injury [46–48]. 

In the current study, an aqueous rooibos extract and RPO were investigated to determine a possible protective effect either individually or combined against *t*-BHP-induced oxidative hepatotoxicity in Wistar rats. HPLC quantification of the aqueous rooibos extract used in this study yielded aspalathin as the major flavonoid present in rooibos which is in accordance with previously published studies [21–24]. Other constituents quantified include orientin, iso-orientin, vitexin, isovitexin, rutin, and trace quantities of quercetin, luteolin, and chrysoeriol. HPLC quantification of the RPO used in this study also yielded isoforms of tocopherols and tocotrienols, as well as *α*- and *β*-carotene in fractions that is in accordance withv previously published works [38, 39].

Evaluation of the total antioxidant capacity (TAC) of food has become a standard, and this is due largely to the renewed interest in health benefits of foods supplements and plants with high antioxidant potentials [75]. While there is no universally accepted measure, the oxygen radical absorbance capacity (ORAC) [54] and the ferric reducing antioxidant power (FRAP) [56] are two of the most popular TAC assays. In the current study, it was observed that the plasma total polyphenol content was significantly reduced in all treatment groups compared to the negative control group. Treatment with *t*-BHP lowered the TAC measured as ORAC in the plasma and the liver. Supplementation with rooibos alone restored the ORAC depletion caused by *t*-BHP treatment in the plasma, with no effect exhibited in the liver. Feeding RPO alone or in combination with rooibos resulted in no net increase in TAC assessed either as ORAC or FRAP in the plasma or liver. In fact, RPO supplementation either alone or in combination tends to lower the TAC of both plasma and liver. Reports on whether supplementation of polyphenol-enriched diets will increase plasma total polyphenols and TAC in rats have been controversial. Apple and pear peels [76] as well as raw and boiled garlic [77] were reported to enhance plasma total polyphenol and TAC while intake of cranberry powder and mango did not produce any such effect [78, 79]. Previous studies, using different rodent models, have reported that rooibos supplementation did not increase the TAC measured as ORAC [25, 80]. Also, Marnewick et al. [28] reported that consumption of six cups of rooibos daily for six weeks did not enhance the plasma antioxidant capacity in adult humans who are at the risk of developing cardiovascular diseases. The assays for antioxidant capacity have been suggested to lack specificity, and their estimates are not likely to indicate any resultant changes in plasma antioxidant capacity [81, 82]. Therefore, this may account for the reason why in the current study, there is no change in plasma antioxidant capacity even in the group consuming rooibos. Furthermore, the plasma antioxidant capacity is a fasting measurement; therefore it may not represent the active antioxidant pool since the half-lives of all the individual compounds, including polyphenols and nonpolyphenols, may fluctuate [28]. In addition, the 12 h fasting period may have a more pronounced effect on the nonphenolic antioxidants making the antioxidant capacity to remain unchanged or diminished regardless of increased polyphenol consumption. Previous reports have indicated that the antioxidant capacity of a compound is dependent upon reaction media [83, 84]. Therefore, an organic-solvent-based ORAC assay would be ideal for RPO which is rich in lipophilic antioxidants. However, fluorescein, used as the fluorescent probe in the ORAC assay, is not sufficiently lipid soluble, and its fluorescence intensity in a nonpolar organic solvent is low. Therefore, this may account for the very low ORAC values observed in the RPO-supplemented groups. 

In recent years, medicinal plants and herbs are getting great attention as important sources of bioactive substances, with health beneficial effects. However, a great limitation to the use of medicinal plants and herbs is the issue of safety and toxicity. Damage to the liver is a widely used indicator of toxicity of medicinal plants and herbs *in vivo *[85, 86]. The aminotransferases (ALT and AST) and LDH are among serum marker enzymes of hepatic function, with their increase in the serum indicating hepatic damage. The supplementation of rooibos, RPO, and/or their combination to normal rats did not result in any toxicity or adverse effects as indicated by the levels of the serum aminotransferases and LDH. Results from this study confirmed *t*-BHP-induced hepatotoxic effects as shown by the significant increase in the activity of ALT, AST and LDH in the serum of *t*-BHP-treated rats. These observations are in accordance with those obtained by previous studies [66, 87–89]. Alanine amino transferase, AST, and LDH are cytoplasmic, and the rise in their serum levels is attributed to damaged structural integrity of the liver and as a result these enzymes are released into the blood circulation after the rupture of the plasma membranes [66, 90]. The *t*-BHP-induced hepatic damage observed was confirmed by histopathology examination of the *t*-BHP-treated rats which revealed severe hepatic degeneration and hepatocyte vacuolation, as well as massive lymphocyte and mononuclear cellular aggregation. Rooibos and RPO supplementation either individually or combined in *t*-BHP-treated rats significantly reduced the elevated levels of ALT, AST, and LDH. The diminished levels of these serum enzymes can be ascribed to a stabilizing effect of the rooibos and RPO phyto-constituents on the plasma membrane of the hepatocytes, as well as repair the damaged hepatic tissues, probably brought about by the stimulation of hepatocellular protein synthesis and accelerated regeneration of the hepatocytes [91]. Histopathological examination of livers from *t*-BHP-treated rats whose diet was supplemented with rooibos and RPO revealed enhanced hepatocellular architecture with slight lymphocyte infiltration, which is a clear manifestation of the hepatoprotective effects of rooibos and RPO. This result is consistent with previous findings that have been reported in different experimental models of rats exposed to other toxicants where rooibos or RPO have been supplemented [72, 74, 92–95]. 

Oxidative stress, manifested as lipid peroxidation, has been implicated in the mechanism of various types of cell injury. It has been hypothesized that one of the principal causes of *t*-BHP-induced liver injury is the formation of lipid peroxides by free radical derivatives (alkoxyl and peroxyl radicals) [68]. In the current study, the *t*-BHP-induced lipid peroxidation was assessed by determining the levels of conjugated dienes (CD) and malondialdehyde (MDA). Plasma, as well as hepatic CD levels, was significantly increased, while hepatic MDA levels were marginally increased by the *t*-BHP treatment. Supplementation with rooibos, RPO, or their combination effectively inhibited this observed increase in the liver. The elevation in CD and MDA levels in the *t*-BHP-treated group in this study may be due to either overproduction of alkoxyl and peroxyl radicals or their accumulation resulting from dysfunction of antioxidant systems during the repeated exposure to *t*-BHP. Previous reports have indicated that rooibos reduced age-related lipid peroxide accumulation (measured as TBARS) in the brain of overage rats consuming the tea for 21 months and inhibited MDA formation in rat tissues and liver microsomal preparations [31, 72, 92, 96]. Recent reports in humans also revealed that rooibos consumption significantly decreased plasma MDA levels in lead factory workers [27] and also significantly lowered plasma CD and MDA levels in adults at the risk for cardiovascular diseases taking 6 cups of rooibos per day for 6 weeks [28]. The ability of rooibos to protect against lipid peroxidation may involve one or more of several different antioxidant properties exhibited by rooibos or synergistic interactions of its different phenolic constituents. The protective effect may be due to the ability of rooibos phenolic constituents not only to bind lipid peroxides, but also their ability to inhibit the lipid peroxidation cascade, either by acting as a sacrificial antioxidant or as a chelator of transition metals that promote lipid peroxidation [12, 37, 97]. Also the protective effect may be associated with the inhibition of cytochrome P450-mediated metabolism of *t*-BHP to active toxic radicals that initiate lipid peroxidation. RPO is a rich source of lipid soluble antioxidants including tocopherols, tocotrienols, and carotenes. Previous reports have highlighted the ability of RPO and its extracts to inhibit lipid peroxidation both *in vitro* and *in vivo*. Wu and Ng [98] reported that a red palm oil extract is able to prevent FeCl_2_-ascorbic acid-induced lipid peroxidation in rat liver and brain homogenates. Cadmium-induced ocular tissue lipid peroxidation was also inhibited by RPO in rabbits [99] while a tocotrienol-rich fraction of RPO was reported to inhibit the level of MDA and protein carbonyl production in the pancreas [100] and the level of MDA + 4-hydroxynonenal in the plasma and aorta [44] of streptozotocin-induced diabetic rats. The potential of RPO to prevent lipid peroxidation induced by *t*-BHP in this study can be attributed to contributions of its lipid antioxidants (tocopherols, tocotrienols, and carotenoids), and this may be rooted in their ability to donate phenolic hydrogen (electrons) to lipid peroxyl radicals [44]. Tocopherols, tocotrienols, and carotenes found in RPO are lipophilic, chain breaking antioxidants which can exert their actions in the hydrophobic lipid core of membranes, thereby protecting the cell membranes from lipid peroxidation induced by *t*-BHP. Supplementation of the combination of rooibos and RPO also reduced *t*-BHP-induced production of CD and MDA in this study; however, the level of reduction was similar to that observed for treated rats consuming either rooibos or RPO alone with no additional protection.

Closely related to lipid peroxidation are the antioxidant enzymes including SOD, CAT, and GPx which are produced by mammalian cells as a defence against ROS generation [12]. Scientific evidence has revealed that oxidative stress mediated by toxic injuries is associated with change in antioxidant enzyme levels and that the specific responses of antioxidant enzymes do not follow set patterns but are stress, tissue, and species specific [101]. In the current study, a marginal increase in the activity of CAT and a significant decrease in the activities of SOD, GR, and GPx in the erythrocyte of *t*-BHP-treated rats were observed. Consumption of rooibos alone or combined with RPO reversed the changes in activities of the antioxidant enzymes induced by *t*-BHP in the erythrocytes. Red palm oil alone, in the diet of the *t*-BHP-treated rats, restored the changes in the activity of CAT, GR, and GPx. In the liver, the activities of CAT and GPx were marginally and significantly decreased, respectively, while GR activity was increased, with SOD unaffected by the *t*-BHP treatment. Rooibos, RPO, or their combination reversed the changes induced in the activities of GR and GPx, but only the combination was effective in augmenting CAT activity. Superoxide dismutase is the first enzyme in the ROS detoxification process, and it converts superoxide radicals to H_2_O_2_. The decrease in SOD activity observed in the erythrocytes of *t*-BHP-treated rats in this study can be adduced to the depletion or inactivation of the enzyme as a result of ROS generation [102], which in turn resulted in the initiation and propagation of lipid peroxidation, which would have contributed to the observed increase in CD and MDA levels discussed earlier. Scavenging of H_2_O_2_-produced by SOD, is the primary role of CAT and the increased CAT activity observed in the erythrocytes of *t*-BHP-treated rats could be a compensatory mechanism attributed to the resultant increased formation of H_2_O_2_ by SOD and/or upregulation of expression of gene encoding for CAT. The fact that both GPx and GR activities were decreased in the erythrocytes leads us to speculate that the metabolism of *t*-BHP in the erythrocytes is via both the cytochrome P450 generation of alkoxyl and peroxyl radicals and the direct detoxification by GSH. The reduction in activity of GPx can be ascribed to its use in catalysing the oxidation of GSH, resulting in the formation of GSSG, which is then reduced to GSH by GR, resulting in NADPH oxidation [7]. In the liver, our observation of a decrease in GPx and an increase in GR activity, while SOD activity was not affected, suggests that organic alkoxyl and peroxyl radicals may not be involved in *t*-BHP-induced oxidative stress in the liver. The activities of these enzymes were modulated to a varying degree in *t*-BHP-treated rats consuming either rooibos, RPO, or their combination. Previously, Marnewick et al. [32] reported that an aqueous rooibos extract modulated the changes observed in the activities of antioxidant enzymes in rats subjected to diethyl-nitrosamine (DEN-) initiated and fumonisin B_1_-(FB_1_-)promoted hepatocarcinogenesis. Another study reported that changes in the activity of SOD and CAT observed in the epididymal sperm of rats subjected to *t*-BHP treatment, were reversed by rooibos supplementation [37]. Another report has also shown that RPO supplementation increased the observed decrease in CAT and SOD activity induced by cadmium in the ocular tissue of rabbit [99]. In the current study, the observed modulation by rooibos, RPO, and/or their combination could be attributed to the natural antioxidants present in them. The flavonoids present in rooibos as well as the tocopherol, tocotrienol, and carotenoids present in RPO may quench free radicals generated by *t*-BHP and/or up- or downregulate the transcription of antioxidant enzyme genes, all which may result in the increase or decrease in their synthesis.

The fact that glutathione (GSH) is involved in defense reactions against oxidative stress as an antioxidant is widely acknowledged [103–105]. Glutathione is the predominant nonenzymatic intracellular antioxidant [106] and participates in the removal of free radicals (including H_2_O_2_, superoxide anion, alkoxyl and peroxyl radicals), maintenance of membrane protein thiols, and it is also a substrate for GPx and GR [107]. Present in the cells in both the reduced (GSH) and oxidized (GSSG) forms, but because of the action of the NADPH-dependent enzyme GR, the cellular content of glutathione is predominantly in favour of GSH under normal physiologic conditions [108]. In agreement with previous reports [6, 7, 88, 89], the current study revealed that *t*-BHP treatment resulted in a reduction in GSH levels both in the erythrocytes and liver, while GSSG level was only increased in the liver. The oxidation of GSH to GSSG is a sensitive marker of oxidative stress and under condition of increased stress, the GSH : GSSG ratio decreases either due to increased GSSG or decreased GSH levels [109]. In this study, *t*-BHP treatment also resulted in a reduction in GSH : GSSG ratio in the liver and erythrocytes. Supplementation with rooibos, RPO and/or their combination reversed the reduction in GSH levels and GSH : GSSG ratio, observed in *t*-BHP-treated rats both in the erythrocytes and liver. Recently, Pantsi et al. [36] reported that a fermented rooibos supplementation restored the decrease in GSH levels and GSH : GSSG ratio in the hearts of rats subjected to ischaemia/reperfusion injury. Similarly, Marnewick et al. [28] also showed that drinking six cups of rooibos per day for six weeks increased the GSH levels and GSH : GSSG ratio in adults at risk for developing cardiovascular disease, while Awoniyi et al. [37] reported that rooibos supplementation in *t*-BHP-treated rats enhance the epididymal sperm GSH levels. The significant increase in GSH level due to rooibos consumption may be attributed to the phenolic antioxidants in rooibos ability to improve the redox/antioxidant status of the cell resulting in an enhanced endogenous detoxification capacity. The polyphenols in rooibos may quench free radicals produced by *t*-BHP, sparring GSH and hence lowering the vulnerability of the cells to further oxidative stress. Another intriguing possibility for the observed GSH increase is that rooibos polyphenols may upregulate the expression of *γ*-glutamylcysteine synthetase (*γ*-GCS), which is the rate limiting enzyme in the synthesis of GSH. Previous studies have shown that polyphenolic compounds from plants increased the *γ*-GCS activity and GSH contents [110–112], although no study has yet been conducted to determine if rooibos or its flavonoids can increase *γ*-GCS mRNA expression. That RPO supplementation restored the observed impairment in the redox status observed in *t*-BHP challenged rat could be ascribed to its vitamin E and carotene constituents. Tocopherols, tocotrienols, and carotenes in RPO are able to quench peroxyl radicals generated by *t*-BHP biotransformation by donating hydrogen from their phenolic hydroxyl group to the peroxyl radical thereby forming a stable radical species [113] and thus spare GSH and protect the cells from oxidative stress. RPO could also increase the biosynthesis of GSH because previous *in vitro* and *in vivo* studies have indicated that *α*-tocopherol [114, 115] and *β*-carotene [116, 117] increased intracellular GSH levels by upregulating the mRNA expression of *γ*-GCS.

One of the hypotheses we set out to investigate in this study is whether cosupplementation of rooibos and RPO to *t*-BHP-challenged rats will result in a synergy of their protective effects. Our results indicated that co-supplementation of rooibos and RPO actually protects against *t*-BHP-induced hepatotoxicity as demonstrated by reduction in level of liver function marker enzymes, inhibition of CD and MDA formation, reversal of changes in antioxidant enzymes, and increase in intracellular GSH level and GSH : GSSG ratio in *t*-BHP-treated rats. However, the level of protection shown is only equal to that of either rooibos or RPO, and thus any synergy in their combine protective effects could not be shown.

## 5. Conclusion

The result of the present investigation suggests that antioxidant-rich rooibos, RPO, and/or their combination, showed efficient protective action against *t*-BHP-induced oxidative hepatotoxicity in rats. This is demonstrated by their ability to (i) reverse the increase in liver function marker enzymes (ALT, AST, and LDH), (ii) prevent lipid peroxidation by reducing the levels of CD and MDA, (iii) modulate changes in activity of antioxidant enzymes, and (iv) restore the redox status by increasing the GSH levels and GSH : GSSG ratio in *t*-BHP-treated rats. The effect observed when rooibos and RPO were supplemented in combination, although protective, was not synergistic. Both rooibos and RPO are rich in antioxidant compounds; therefore, the effects observed for each extract are proposed to be as a result of synergistic interaction of all the compounds in each extract. The protective biochemical function of naturally occurring antioxidants in biological system and their mechanisms of action are gaining more attention. This study therefore provides biological evidence supporting the use of rooibos and RPO as an adjuvant therapy for the prevention and treatment of liver disorders; however, a series of well-controlled clinical intervention studies are needed to explore this possibility further.

## Figures and Tables

**Table 1:** Animal treatment and experimental design.

Groups	Treatments		
	RTE (2% w/v)	RPO (7 g/kg diet)	*t*-BHP (30 *µ*mol/100 g body weight)
Negative control (water)	−	−	−
Positive control (*t*-BHP)	−	−	+
RTE	+	−	−
RPO	−	+	−
RTE + RPO	+	+	−
RTE + *t*-BHP	+	−	+
RPO + *t-*BHP	−	+	+
RTE + RPO + *t*-BHP	+	+	+

*t*-BHP: *tert*-butyl hydroperoxide, RTE: aqueous rooibos extract, RPO: red palm oil.

**Table 2:** Phenolic content and antioxidant capacity of aqueous rooibos (2%, w/v) extract.

Soluble solids (mg/mL)	Total phenolic content (mg gallic acid equivs/mg soluble solids)	Flavonol content (mg quercetin equivs/mg soluble solids)	Flavanol content (mg catechin equivs/mg soluble solids)	FRAP (*µ*mol AAE/mL)	TEAC (*µ*mol TE/mL)	ORAC (*µ*mol TE/mL)
2.743 ± 0.26	0.303 ± 0.006	0.159 ± 0.004	0.058 ± 0.002	4.90 ± 0.35	5.22 ± 0.22	14.72 ± 1.57

Values are mean ± SEM. Soluble solids content is a mean of 15 determinations while other parameters are mean of 6 determinations. AAE: ascorbic acid equivalent, TE: trolox equivalent, FRAP: ferric reducing ability of the plasma, ORAC: oxygen radical absorbance capacity, TEAC: trolox equivalent antioxidant capacity.

**Table 3:** HPLC quantification of flavonoids in aqueous rooibos tea extract consumed by rats.

Phenolic compound	Concentration (*µ*g/mL)	% of Soluble solids	Daily intake (mg/100 g BW)
Aspalathin	28.32 ± 1.65	1.03 ± 0.06	0.24 ± 0.01
Orientin	16.94 ± 1.67	0.62 ± 0.06	0.15 ± 0.01
Isoorientin	23.64 ± 2.34	0.86 ± 0.09	0.20 ± 0.02
Vitexin	6.06 ± 0.61	0.22 ± 0.02	0.05 ± 0.01
Isovitexin	6.50 ± 0.68	0.24 ± 0.02	0.06 ± 0.01
Hyperoside/rutin	14.55 ± 1.30	0.53 ± 0.05	0.13 ± 0.01
Quercetin	0.89 ± 0.11	0.03 ± 0.003	0.01 ± 0.001
Luteolin	0.22 ± 0.03	0.01 ± 0.001	Trace amount
Chrysoeriol	0.23 ± 0.02	0.01 ± 0.001	Trace amount

Soluble solids (mg/mL) 2.74 ± 0.26			

Values are mean ± SEM (*n* = 4). BW (body weight).

**Table 4:** Fluid and phenolic intake of rats fed aqueous rooibos tea extract for a period of 8 weeks.

Treatment	Water/rooibos intake/day/100 g BW (mL)	Total phenolic intake (mg gallic acid equivs/day/100 g BW)	Flavonol intake (mg quercetin equivs/day/100 g BW)	Flavanol intake (mg catechin equivs/day/100 g BW)
Negative control (water)	9.29 ± 0.25^a^	ND	ND	ND
Positive control (*t*-BHP)	8.53 ± 0.20^a^	ND	ND	ND
RTE	8.90 ± 0.26^a^	7.38 ± 0.21^a^	3.87 ± 0.11^a^	1.41 ± 0.04^a^
RPO	8.83 ± 0.21^a^	ND	ND	ND
RTE + RPO	8.81 ± 0.20^a^	7.31 ± 0.17^a^	3.83 ± 0.09^a^	1.39 ± 0.03^a^
RTE + *t*-BHP	8.99 ±0.32^a^	7.45 ± 0.26^a^	3.91 ± 0.1^a^	1.42 ± 0.05^a^
RPO + *t*-BHP	8.63 ± 0.33^a^	ND	ND	ND
RTE + RPO + *t*-BHP	7.72 ± 0.15^b^	6.40 ± 0.13^b^	3.36 ± 0.07^b^	1.22 ± 0.02^b^

ND: not determined. Calculations of the total phenolic, flavonol, and flavanol intakes were calculated based on the soluble solid intake obtained from the average rooibos consumption per day. Values are mean ± SEM (*n* = 10). Mean followed by different superscript is significantly different at *P* < 0.05. RTE: aqueous rooibos extract, RPO: red palm oil, *t*-BHP: *tert*-butyl hydroperoxide.

**Table 5:** Daily intakes, vitamin E, and carotene content of RPO.

Constituent	Concentration (*µ*g/g RPO)	Daily intake (*µ*g)
*α*-Tocotrienol	102.36 ± 0.68	17.91 ± 0.12
*β*/*γ*-Tocotrienol	227.48 ± 1.22	39.81 ± 0.21
*δ*-Tocotrienol	56.46 ± 0.69	9.88 ± 0.12
*α*-Tocopherol	71.28 ± 1.03	12.47 ± 0.18
*β*/*γ*-Tocopherol	6.20 ± 0.42	1.09 ± 0.07
*δ*-Tocopherol	20.70 ± 0.74	3.62 ± 0.13
*α*-Carotene	23.74 ± 0.52	4.15 ± 0.09
*β*-Carotene	29.34 ± 1.30	5.14 ± 0.23

Values are mean ± SEM (*n* = 5).

**Table 6:** Effects of rooibos and RPO consumption on body weight gain, liver weight, and relative liver weight in all experimental rats.

Treatment	Body weight gain (g)	Liver weight (g)	Relative liver weight (%)
Negative control (water)	150.64 ± 3.56	10.78 ± 0.30	2.96 ± 0.10
Positive control (*t*-BHP)	127.90 ± 6.48	10.90 ± 0.47	3.12 ± 0.09
RTE	150.15 ± 8.67	11.93 ± 0.47	3.14 ± 0.10
RPO	133.54 ± 5.26	10.45 ± 0.37	2.82 ± 0.07
RTE + RPO	138.25 ± 7.15	11.27 ± 0.40	2.83 ± 0.07
RTE + *t*-BHP	152.33 ± 5.54	12.47 ± 0.51	3.23 ± 0.11
RPO + *t*-BHP	134.80 ± 7.37	10.19 ± 0.32	2.65 ± 0.05^#^
RTE + RPO + *t*-BHP	138.88 ± 3.08	11.51 ± 0.34	2.81 ± 0.07

Values are mean ± SEM (*n* = 10). ^#^Significantly different versus positive control (*P* < 0.05). RTE: aqueous rooibos extract, RPO: red palm oil, *t*-BHP: *tert*-butyl hydroperoxide.

**Table 7:** Effects of aqueous rooibos, RPO, or their combination on total polyphenol content and antioxidant capacity of plasma and liver of all experimental rats.

Treatment	Plasma			Liver	
	Total polyphenol content (mg GAE/L)	ORAC (*µ*mol TE/L)	FRAP (*µ*mol AAE/L)	ORAC (*µ*mol TE/g tissue)	FRAP (*µ*mol AAE/g tissue)
Negative control (water)	65.27 ± 2.71	1934.32 ± 101.82	204.85 ± 31.02	15.20 ± 0.39	2.01 ± 0.06
Positive control (*t*-BHP)	51.11 ± 1.48*	1535.97 ± 50.60*	185.81 ± 11.15	11.51 ± 0.59*	1.99 ± 0.05
RTE	55.38 ± 2.05*	2082.34 ± 88.11	291.75 ± 52.82	14.29 ± 1.23	2.08 ± 0.07
RPO	45.97 ± 1.33*	1284.86 ± 42.14*	210.96 ± 21.18	13.18 ± 0.39*	2.16 ± 0.04
RTE + RPO	56.43 ± 2.60*	1437.41 ± 90.66*	207.84 ± 14.48	14.50 ± 0.75	2.04 ± 0.05
RTE + *t*-BHP	55.66 ± 3.92*	1721.08 ± 153.85^#^	243.04 ± 28.98	9.99 ± 1.04*	2.21 ± 0.05
RPO + *t*-BHP	54.53 ± 2.98*	1296.03 ± 74.88*	207.08 ± 19.47	10.00 ± 0.68*	2.11 ± 0.04
RTE + RPO + *t*-BHP	54.37 ± 2.43*	1505.31 ± 95.46*	203.91 ± 18.92	10.65 ± 0.85*	2.07 ± 0.04

Values are mean ± SEM of 7–10 rats per group. *Significantly different from negative control group (*P* < 0.05). ^#^Significantly different from positive control group (*P* < 0.05). ORAC: oxygen radical absorbance capacity, FRAP: ferric reducing ability of plasma, RTE: aqueous rooibos extract, RPO: red palm oil, *t*-BHP: *tert*-butyl hydroperoxide, AAE: ascorbic acid equivalent, GAE: gallic acid equivalent, TE: trolox equivalent.

**Table 8:** Effects of aqueous rooibos extract, RPO, and/or their combination on antioxidant enzymes activities in erythrocyte and liver of all experimental rats.

Treatment	Erythrocytes				Liver			
	CAT	GR	SOD	GPx	CAT	GR	SOD	GPx
Negative (water)	0.64 ± 0.05	0.56 ± 0.06	5.45 ± 1.71	1.76 ± 0.17	198.90 ± 9.17	17.03 ± 0.50	37.46 ± 4.05	28.41 ± 1.84
Positive (*t*-BHP)	0.87 ± 0.11**	0.21 ± 0.05*	1.72 ± 0.59*	0.85 ± 0.23*	178.31 ± 5.63**	20.14 ± 1.14*	46.98 ± 3.18	22.10 ± 1.04*
RTE	0.22 ± 0.03*	0.85 ± 0.14	5.96 ± 0.76	1.74 ± 0.14	202.88 ± 5.08	16.16 ± 0.50	38.66 ± 3.04	35.01 ± 1.44*
RPO	0.41 ± 0.02*	0.44 ± 0.05	2.54 ± 0.34	1.62 ± 0.37	197.79 ± 6.23	16.47 ± 0.40	31.14 ± 1.77	29.91 ± 1.19
RTE + RPO	0.20 ± 0.02*	0.42 ± 0.09	7.42 ± 1.56	2.02 ± 0.22	233.26 ± 4.34*	16.43 ± 0.70	34.35 ± 3.49	42.40 ± 4.02*
RTE + *t*-BHP	0.30 ± 0.02^∗#^	0.44 ± 0.05^#^	4.12 ± 0.89^#^	1.67 ± 0.14^#^	195.51 ± 6.39	16.25 ± 0.30^#^	52.10 ± 3.34	31.45 ± 1.93^#^
RPO + *t*-BHP	0.43 ± 0.17^∗#^	0.43 ± 0.05^#^	1.13 ± 0.25	1.55 ± 0.09^#^	186.11 ± 6.01	16.38 ± 0.40^#^	38.14 ± 1.86	32.75 ± 2.28^#^
RTE + RPO + *t*-BHP	0.45 ± 0.05^∗#^	0.40 ± 0.07^#^	7.21 ± 1.20^#^	2.10 ± 0.41^#^	199.37 ± 8.20^#^	16.37 ± 0.40^#^	36.19 ± 2.39	37.64 ± 3.10^#^

Values in columns are mean ± SEM for 7–10 rats per group. *Significantly different from negative control (*P* < 0.05). **Marginally different from negative control (*P* < 0.1). ^#^Significantly different from positive control (*P* < 0.05). CAT: catalase, *µ*mol H_2_O_2_ consumed/min/*µ*g protein in the erythrocyte or *µ*mol H_2_O_2_ consumed/min/mg protein in the liver, GR: glutathione reductase, *µ*mol NADPH oxidized/min/*µ*g protein in the erythrocyte or *µ*mol NADPH oxidized/min/mg protein in the liver, SOD: superoxide dismutase, U/*µ*g protein in erythrocyte or U/mg protein in the liver, GPx: glutathione peroxidase, nmol NADPH oxidized/min/*µ*g protein in the erythrocyte or nmol NADPH oxidized/min/mg protein in the liver, RTE: aqueous rooibos extract, RPO: red palm oil, *t*-BHP: *tert*-butyl hydroperoxide.

**Table 9:** Effects of aqueous rooibos extract, RPO, and/or their combination on markers of lipid peroxidation in the plasma and liver of all experimental rats.

Treatment	Plasma		Liver	
	CD (nmol/L)	MDA (*µ*mol MDA/L)	CD (nmol/g tissue)	MDA (*µ*mol MDA/g tissue)
Negative control (water)	71.67 ± 2.43	2.44 ± 0.09	7.29 ± 0.15	0.37 ± 0.09
Positive control (*t*-BHP)	89.75 ± 1.30*	2.72 ± 0.16	8.98 ± 0.12*	0.62 ± 0.04**
RTE	72.60 ± 1.24	2.55 ± 0.07	7.54 ± 0.17	0.10 ± 0.01*
RPO	108.89 ± 13.4*	2.52 ± 0.11	7.50 ± 0.12	0.10 ± 0.004*
RTE + RPO	102.11 ± 4.91*	2.49 ± 0.07	7.44 ± 0.17	0.11± 0.003*
RTE + *t*-BHP	101.69 ± 5.18^#^	2.61 ± 0.11	7.56 ± 0.21^#^	0.10 ± 0.01^∗#^
RPO + *t*-BHP	103.67 ± 4.67^#^	2.45 ± 0.13	7.98 ± 0.22^#^	0.16 ± 0.06^∗#^
RTE + RPO + *t*-BHP	106.83 ± 2.15^#^	2.42 ± 0.09	7.79 ± 0.11^#^	0.11 ± 0.01^∗#^

Values in columns are mean ± SEM of 8–10 rats per group. *Significantly different from negative control (*P* < 0.05). **Marginally different from negative control (*P* < 0.1). ^#^Significantly different from positive control (*P* < 0.05). CD: conjugated diene, MDA: malondialdehyde, RTE: aqueous rooibos extract, RPO: red palm oil, *t*-BHP: *tert*-butyl hydroperoxide.

**Table 10:** Effects of aqueous rooibos extract, RPO, and/or their combination on glutathione status in the erythrocyte and liver of all experimental rats.

Treatment	Erythrocyte			Liver		
	GSH (*µ*mol/*µ*g protein)	GSSG (*µ*mol/*µ*g protein)	GSH:GSSG	GSH (*µ*mol/g wet liver)	GSSG (*µ*mol/g wet liver)	GSH:GSSG
Negative control (water)	0.210 ± 0.037	0.121 ± 0.012	1.70 ± 0.20	6.13 ± 0.09	0.46 ± 0.08	18.52 ± 1.57
Positive control (*t*-BHP)	0.064 ± 0.012*	0.150 ± 0.014	0.41 ± 0.06*	3.84 ± 0.39*	0.56 ± 0.09	8.54 ± 1.63*
RTE	0.190 ± 0.025	0.126 ± 0.011	1.59 ± 0.25	7.82 ± 0.47*	0.23 ± 0.03*	32.54 ± 4.35*
RPO	0.195 ± 0.024	0.109 ± 0.005	1.75 ± 0.16	5.89 ± 0.39	0.23 ± 0.04*	33.05 ± 6.48*
RTE + RPO	0.321 ± 0.027*	0.117 ± 0.003	2.72 ± 0.18*	8.61 ± 0.52*	0.22 ± 0.04*	54.27 ± 10.35*
RTE + *t*-BHP	0.159 ± 0.026^#^	0.112 ± 0.005	1.42 ± 0.20^#^	7.28 ± 0.52^∗#^	0.32 ± 0.04^#^	24.76 ± 2.96^#^
RPO + *t*-BHP	0.182 ± 0.023^#^	0.109 ± 0.004	1.65 ± 0.19^#^	5.84 ± 0.29^#^	0.14 ± 0.01^#^	41.47 ± 2.58^#^
RTE + RPO + *t*-BHP	0.240 ± 0.022^#^	0.126 ± 0.008	1.89 ± 0.13^#^	7.72 ± 0.27^#^	0.37 ± 0.03^#^	22.92 ± 3.24^#^

Values are mean ± SEM of 8–10 rats per group. *Significantly different from negative control (*P* < 0.05). ^#^Significantly different from positive control (*P* < 0.05). GSH: reduced glutathione, GSSG: oxidised glutathione, RTE: aqueous rooibos extract, RPO: red palm oil, *t*-BHP: *tert*-butyl hydroperoxide.
